# Etiology and factors associated with urogenital fistula among women who have undergone cesarean section: a cross-sectional study

**DOI:** 10.1186/s12884-023-05357-3

**Published:** 2023-01-23

**Authors:** Raha Maroyi, Madeline K. Moureau, Heidi W. Brown, Rane Ajay, Gloire Byabene, Denis M. Mukwege

**Affiliations:** 1 Department of Urogynecology, Panzi General Referral Hospital, Bukavu, Democratic Republic of the Congo; 2 Faculty of Medicine, Evangelical University in Africa, Bukavu, Democratic Republic of the Congo; 3 Mushununu, Panzi, Bukavu, Democratic Republic of the Congo; 4 Department of Obstetrics and Gynecology, University of Wisconsin School of Medicine and Public Health, Madison, USA; 5 Department of Obstetrics and Gynecology, James Cook University, Townsville, Australia; 6 Department of General Surgery, Panzi General Referral Hospital, Bukavu, Democratic Republic of the Congo; 7 Department of Gynecology and Obstetrics, Panzi General Referral Hospital, Bukavu, Democratic Republic of the Congo

**Keywords:** Female, Obstetric fistula, Cesarean section, Obstructed labor, Fetal demise, The Democratic Republic of the Congo

## Abstract

**Background:**

The prevalence and impact of fistulas are more common in developing countries with limited access to emergency obstetric care. As a result, women in these settings often experience adverse psychosocial factors. The purpose of this study was to describe the characteristics of Congolese women who developed urogenital fistula following Cesarean sections (CS) to determine the characteristics associated with two etiologies: (1) prolonged obstructed labor; and (2) a complication of CS following obstructed labor.

**Methods:**

We performed a cross-sectional study on abstracted data from all patients with urogenital fistula following CS who received care during a surgical campaign in a remote area of the Democratic Republic of the Congo (DRC). Descriptive analyses characterized patients with fistula related to obstructed labor versus CS. Univariate and multivariate logistic regression models identified factors associated with obstetric fistula after cesarean delivery following obstructed labor. Variables were included in the logistic regression models based upon biological plausibility.

**Results:**

Among 125 patients, urogenital fistula etiology was attributed to obstructed labor in 77 (62%) and complications following CS in 48 (38%). Women with a fistula, attributed to obstructed labor, developed the fistula at a younger age (*p* = .04) and had a lower parity (*p* = .02). Attempted delivery before arriving at the hospital was associated with an increased risk of obstetric fistula after cesarean delivery following obstructed labor (*p* < .01).

**Conclusion:**

CS are commonly performed on women who arrive at the hospital following prolonged obstructed labor and fetal demise, and account for almost 40% of urogenital fistula. Obstetric providers should assess maternal status upon arrival to prevent unnecessary CS and identify women at risk of developing a fistula.

## Introduction

An obstetric fistula is defined as an opening between the genital tract and bladder or rectum that results in chronic urinary or fecal leakage [1, 2]. Obstetric fistulas are estimated to affect between 50,000 to 100,000 women globally each year, with approximately 2 million women currently living with an obstetric fistula [2]. Unfortunately, estimating the prevalence of obstetric fistulas in the Democratic Republic of the Congo (DRC) is challenging due to the majority of women living in remote areas, political instability, and lack of health infrastructure [3]. With limited healthcare resources to repair obstetric fistula, women in these settings often experience chronic leakage leading to negative societal impacts including divorce, loss of income, and social isolation [1, 4]. As a result of the prevalence and negative impact of obstetric fistula on women in developing countries, the United Nations has called for the end of obstetric fistulas by 2030 as part of the Sustainable Development Goals [5].

Prolonged obstructed labor is the leading cause of obstetric fistulas globally and is more common in settings with limited access to emergency obstetric care [6]. In the absence of skilled medical care, prolonged obstructed labor can continue for days, resulting in fetal death and ischemic necrosis of the genital tract that can lead to the development of an obstetric fistula [7]; the co-occurrence of obstetric fistula and fetal death has previously been described as a double burden of tragedy [8]. When women with an obstetric fistula seek care, they often report their fistula developed after undergoing a Cesarean section (CS) [9]. Recently, studies have found that the prevalence of iatrogenic fistula associated with CS is increasing as CS rates increase; this suggests that when women present to a tertiary care facility following prolonged obstructed labor, the CS itself may increase morbidity and create additional burden [9, 10].

We sought to describe the characteristics of Congolese women who developed urogenital fistula following CS to determine characteristics associated with two different fistula etiologies: (1) obstructed labor; and (2) CS following obstructed labor.

## Methods

### Study design and setting

In 2011, a surgical outreach program was developed by Panzi General Reference Hospital in the South Kivu province of the DRC [11]. The program was initiated to provide specialized fistula care to women in remote, underserved areas who are not able to travel to Panzi Hospital for treatment. Each year, approximately 700 women receive surgical fistula repair through the surgical outreach program.

### Data collection

We conducted a cross-sectional study on patients who developed urogenital fistula following CS and received care from the surgical outreach program in 2016. Patients were provided informed consent and those who consented to participate in the study were interviewed regarding their medical history. Data collected during the interview at the time of surgical repair included (1) patient demographics (age, profession, education level, and marital status); (2) obstetric history (parity, characteristics of labor); (3) characteristics of fistula; and (4) medical conditions. The type and location of the fistula were confirmed during pre and postoperative clinical exams and classified according to the anatomical location: ureterovaginal (located on the left or right ureter); vesicouterine (intracervical); and vesicovaginal (low / mid vagina or apical / juxtacervical). At the time of surgical fistula repair, urogenital fistula etiology was designated as either related to (1) prolonged obstructed labor; or (2) surgical complication of CS.

### Statistical analysis

Descriptive analyses characterized patients with urogenital fistula related to obstructed labor versus CS. Univariate and multivariate logistic regression models identified factors associated with obstetric fistula after cesarean delivery following obstructed labor. Variables were included in the logistic regression models based upon biological plausibility: age, parity, days in labor, whether labor started at the hospital, and whether delivery was attempted before arrival to the hospital. A *p*-value of less than 0.05 was considered statistically significant. Analyses were performed using SPSS 26.0.

### Ethical considerations

This study was approved by the DRC National Health Ethics Committee (UCB/CIE/NC/10/2013). All study procedures adhered to institutional ethical standards and the Declaration of Helsinki and its later amendments. Upon enrollment in this study, informed consent was obtained from participants.

## Results

Among 125 patients who consented to participate, the median age was 30 years, with 76% being farmers, 66% being married, and 46% having never received formal education at the time of surgical repair (Table 1). The etiology of the urogenital fistula was attributed to obstructed labor in 77 (62%) and to complications of CS in 48 (38%). Women whose fistula was attributed to complications of CS were more likely to be single, while women whose fistula was related to obstructed labor were more likely to be married or widowed. There were no other significant demographic differences between the two groups. Overall, 42% of women reported a separation or divorce related to their CS, fetal demise, or fistula (46% among women with fistula due to CS and 39% with fistula due to CS, *p* = 0.45).

**Table 1:** Sample Description Stratified by Etiology of Fistula

Characteristic	Total sample (*N* = 125)	Etiology of Fistula		*P*-Value
		Cesarean Section (*N* = 48)	Obstructed Labor (*N* = 77)	
Age in years (median, IQR)	30 (24.5–42.5)	32 (26.25–45)	30 (24–40.5)	.209
Education level				.540
None	58 (46.4)	21 (43.8)	37 (48.1)	
Some primary	44 (35.2)	16 (33.3)	28 (36.4)	
Completed primary	5 (4.0)	2 (4.2)	3 (3.9)	
Some secondary	15 (12.0)	9 (18.8)	6 (7.8)	
Completed secondary or above	3 (2.4)	0 (0)	3 (3.9)	
Profession				.323
Farmer	95 (76.0)	34 (70.8)	61 (79.2)	
Housekeeper	19 (15.2)	8 (16.7)	11 (14.3)	
Teacher	1 (0.8)	0 (0)	1 (1.3)	
Merchant	5 (4.0)	4 (8.3)	1 (1.3)	
Other	5 (4.0)	2 (4.2)	3 (3.9)	
Marital status				.024
Married	82 (65.6)	29 (60.4)	53 (68.8)	
Single	5 (4.0)	5 (10.4)	0 (0)	
Divorced/separated	29 (23.2)	12 (25.0)	17 (22.1)	
Widowed	9 (7.2)	2 (4.2)	7 (9.1)	

*IQR* Inter-quartile range

Table 2 describes fistula characteristics stratified by fistula etiology. The median age at presentation was 30 years (range 16–80) and the median age at fistula development was 24 years (range 11–51). More than half of the participants had a fistula for 6 years or more prior to undergoing surgical correction.

**Table 2:** Characteristics Related to Urogenital Fistula Stratified by Etiology of Fistula

Characteristic	Total sample (*N* = 125)	Etiology of Fistula		*P*-Value
		Cesarean Section (*N* = 48)	Obstructed Labor (*N* = 77)	
Age when fistula developed (median, IQR)	24 (18–30.5)	26 (19.25–36.5)	23 (18–30)	**.043**
Years with fistula (median, IQR)	6 (2–15)	4.5 (1–15.75)	6 (2–13.5)	.614
Years with fistula				.368
0–5	61 (48.8)	26 (54.2)	35 (45.5)	
6–15	35 (28.0)	10 (20.8)	25 (32.5)	
15 +	29 (23.2)	12 (25.0)	17 (22.1)	
Parity at fistula (median, IQR)	3 (1.5–5)	4 (2–5)	2 (1–4.5)	**.020**
Days in labor (median, IQR)	2 (1–3)	2 (1–3)	2 (1–3)	.318
Where labor started				**.002**
Home	55 (44.0)	13 (27.1)	42 (54.5)	
Health center	45 (36.0)	19 (39.6)	26 (33.8)	
Hospital	25 (20.0)	16 (33.3)	9 (11.7)	
Individual that helped in labor				**.003**
Family member	55 (44.0)	13 (27.1)	42 (54.5)	
Medical worker	70 (56.0)	35 (72.9)	35 (45.5)	
Delivery attempted before arriving at hospital	100 (80.0)	32 (66.7)	68 (88.3)	**.003**
Type of Cesarean Section				**.001**
Fetal extraction	119	42 (87.5)	77 (100.0)	
Cesarean Section (fetus alive)	(95.2) 6 (4.8)	6 (12.5)	0 (0)	
Number of days post CS that urine leakage occurred (median, IQR)	2 (1–4)	2 (1–7)	2 (1–3)	.055
Number of days post CS that urine leakage occurred – grouped				**.005**
1–2	74 (59.2)	25 (52.1)	49 (63.6)	
3–6	27 (21.6)	7 (14.6)	20 (26.0)	
7 +	24 (19.2)	16 (33.3)	8 (10.4)	
Fistula type and location				** < .001**
Ureterovaginal				
Left	10 (8.0)	10 (20.8)	0 (0)	
Right	3 (2.4)	3 (6.3)	0 (0)	
Vesicouterine (intracervical)	35 (28.0)	35 (72.9)	0 (0)	
Vesicovaginal				
Mid or low vagina	38 (30.4)	0 (0)	38 (49.4)	
Apical (juxtacervical)	39 (31.2)	0 (0)	39 (50.6)	
Surgical route				** < .001**
Abdominal	49 (39.2)	46 (95.8)	3 (3.9)	
Vaginal	76 (60.8)	2 (4.2)	74 (96.1)	

*IQR* Inter-quartile range, *CS* Cesarean section

Women with a fistula attributed to obstructed labor developed the fistula at a significantly younger age than women with a fistula related to CS at the time of delivery (23 versus 26 years, *p* = 0.04) and had a lower parity as well (median 2 versus 4, *p* = 0.02). While both groups were in labor for a similar duration, women whose fistula was attributable to obstructed labor were more likely to have started labor at home (*p* = 0.002) and attempted delivery before arriving at the hospital (*p* = 0.003). Women whose fistula was related to the CS were more likely to develop urine leakage delayed from delivery (*p* = 0.005). The vast majority of fistulas related to obstructed labor were repaired vaginally, while fistulas related to CS were repaired abdominally (*p* < 0.001).

Factors associated with obstetric fistula after cesarean delivery following obstructed labor are displayed in Table 3. On univariate logistic regression analysis, age when the fistula developed (*p* = 0.02) and parity when the fistula developed (*p* = 0.04) were associated with decreased risk of obstetric fistula after cesarean delivery following obstructed labor; attempted delivery prior to arriving at the hospital (*p* = 0.005) was associated with increased risk. Only attempted delivery before arrival to the hospital was significant in the multivariate logistic regression model (*p* = 0.003).

**Table 3:** Factors associated with obstetric fistula after cesarean delivery following obstructed labor

Variable	Odds Ratio (95% CI)	Adjusted Odds Ratio (95% CI)
Age when fistula developed	**.953 (.915-.992)**	.974 (.928–1.022)
Parity when fistula developed	**.862 (.747-.995)**	.922 (.778–1.094)
Days in labor during delivery fistula developed	1.169 (.855–1.597)	1.146 (.865–1.518)
Labor started at hospital	.778 (.369–1.642)	.449 (.187–1.080)
Delivery attempted before arriving at hospital	**3.778 (1.508–9.464)**	**5.156 (1.773–14.998)**

*CI* Confident interval

## Discussion

In this study of 125 patients with a history of urogenital fistula following CS in the DRC, over one-third had a fistula attributable to their CS and all of these fistulas required abdominal surgery to repair. Women with a fistula attributable to obstructed labor were more likely to have attempted delivery prior to arriving at the hospital and were more likely to undergo vaginal surgery for repair.

Previous studies conducted in Sub-Saharan Africa have found that obstetric fistulas are more common in uneducated women [12, 13]. In 2013, Hawkins et al. reported that 45% of women in Kenya who developed an obstetric fistula had no prior education, similar to our findings in this study that 46% of women with an obstetric fistula had no prior education [12]. With more than half of the participants waiting six years or more to seek treatment for their obstetric fistula, future analyses should explore women’s knowledge of obstetric fistulas and their treatment options in the DRC.

Of significance, 42% of women in our study reported experiencing a separation or divorce as a consequence of their urogenital fistula, obstructed labor, or CS. Khisa et al. previously analyzed the experiences of women with obstetric fistula in Kenya and found similar adverse societal impacts, specifically noting the high prevalence of divorce, stigma, and psychological trauma [14]. Unfortunately, even after fistula repair, women continue to experience social isolation from their families and communities and are referred to as “spoiled and not accepted,” indicating that even if their fistula is repaired, residual effects may persist [14, 15].

While the association between younger age at marriage and first birth and development of obstetric fistula has been documented in other sub-Saharan African countries [16–18], the median age at symptom development in our cohort was relatively old (24 years). It is possible that our unique sample of patients who participated in a surgical outreach campaign accounts for this difference.

A previous literature review concluded that obstetric fistulas can be reduced if women seek timely care in labor [19]. Of note, the women in this study were from remote, underserved areas, which may explain why women attempted delivery prior to arriving at the hospital. Importantly, even when women arrived at the hospital, they were likely to undergo CS for a stillborn neonate.

Of the 125 patients in this study that experienced prolonged obstructed labor, 119 (95%) resulted in fetal demise. The fistula etiology, among women who had a stillbirth, was later attributed to CS in 42 (35%) and obstructed labor in 77 (65%); a fistula attributed to CS on ischemic tissues after prolonged obstructed labor shows a peculiar pattern of development compared to fistula attributed to vaginal extraction. Similar findings have been reported by Ngongo et al. in a retrospective review of nine sub-Saharan African countries (not including the DRC) [9]. Ngongo et al. found that in women delivering a stillborn baby, CS increased while assisted vaginal delivery decreased [9]. This common finding is likely due to the lack of resources in hospitals to perform alternative procedures and inadequate training for obstetric providers and staff on alternative fetal extraction options [20, 21]. Instead of potentially inducing a triple burden of tragedy on women, including fetal demise, CS, and urogenital fistula, future initiatives need to (1) focus on training obstetric providers and staff on alternative fetal extraction options; (2) train providers how to assess maternal status upon arrival at the hospital; and (3) build healthcare infrastructure allowing for supplies to be adequately acquired by healthcare facilities.

With limited research examining the etiology and factors associated with urogenital fistula in women from the DRC, this study provides meaningful insight about factors associated with the development of urogenital fistula in low-resource settings. Importantly, complex fistulas develop following CS after obstructed labor. Although the cross-sectional methodology used for this analysis limits the conclusions we are able to make, this study highlights the increased morbidity associated with CS among women with obstructed labor. Namely, among women who developed fistulas following obstructed labor, more than one-third have complex fistulas related to cesarean delivery. Future studies should explore barriers experienced by birthing people from the DRC and other sub-Saharan African countries. Additionally, research should explore how to prevent unnecessary CS in women with obstructed labor and fetal demise to prevent the development of more complicated obstetric fistula.

## Conclusion

In conclusion, in the DRC, CS are commonly performed on women who arrive at the hospital following prolonged obstructed labor and fetal demise. These women are already at high risk for urogenital fistula, and CS increases the risk of complex fistula requiring an abdominal approach to repair. We propose that obstetric providers conduct an assessment on women who present with fetal demise to determine whether patients are at risk of developing an obstetric fistula. Obstetric providers can then determine if a vaginal delivery or CS is more appropriate for the patient in an effort to reduce maternal morbidity. Training obstetric providers and staff to assess maternal status upon arrival may prevent unnecessary CS and decrease women’s risk of developing a urogenital fistula.

## Data Availability

The datasets used and/or analyzed during the current study are available from the corresponding author on reasonable request.

## Abbreviations

DRC: Democratic Republic of the Congo
CS: Cesarean section
